# Plasma DNA methylation: a potential biomarker for stratification of liver fibrosis in non-alcoholic fatty liver disease

**DOI:** 10.1136/gutjnl-2016-311526

**Published:** 2016-03-21

**Authors:** Timothy Hardy, Mujdat Zeybel, Christopher P Day, Christian Dipper, Steven Masson, Stuart McPherson, Elsbeth Henderson, Dina Tiniakos, Steve White, Jeremy French, Derek A Mann, Quentin M Anstee, Jelena Mann

**Affiliations:** 1 Fibrosis Laboratories, Institute of Cellular Medicine, Newcastle University, Newcastle upon Tyne, UK; 2 Department of Gastroenterology and Hepatology, Newcastle upon Tyne Hospitals NHS Foundation Trust, Newcastle upon Tyne, UK; 3 School of Medicine, Koç University, Istanbul, Turkey; 4 Department of Cellular Pathology, Newcastle upon Tyne Hospitals NHS Foundation Trust, Newcastle upon Tyne, UK; 5 Department of Hepatobiliary Surgery, Newcastle upon Tyne Hospitals NHS Foundation Trust, Newcastle upon Tyne, UK

**Keywords:** NONALCOHOLIC STEATOHEPATITIS, HEPATIC FIBROSIS, MOLECULAR GENETICS, MOLECULAR PATHOLOGY, PPAR GAMMA

## Abstract

**Objective:**

Liver biopsy is currently the most reliable way of evaluating liver fibrosis in patients with non-alcoholic fatty liver disease (NAFLD). Its inherent risks limit its widespread use. Differential liver DNA methylation of peroxisome proliferator-activated receptor gamma (PPARγ) gene promoter has recently been shown to stratify patients in terms of fibrosis severity but requires access to liver tissue. The aim of this study was to assess whether DNA methylation of circulating DNA could be detected in human plasma and potentially used to stratify liver fibrosis severity in patients with NAFLD.

**Design:**

Patients with biopsy-proven NAFLD and age-matched controls were recruited from the liver and gastroenterology clinics at the Newcastle upon Tyne Hospitals NHS Foundation Trust. Plasma cell-free circulating DNA methylation of PPARγ was quantitatively assessed by pyrosequencing. Liver DNA methylation was quantitatively assessed by pyrosequencing NAFLD explant tissue, subjected to laser capture microdissection (LCM). Patients with alcoholic liver disease (ALD) were also subjected to plasma DNA and LCM pyrosequencing.

**Results:**

26 patients with biopsy-proven NAFLD were included. Quantitative plasma DNA methylation of PPARγ stratified patients into mild (Kleiner 1–2) and severe (Kleiner 3–4) fibrosis (CpG1: 63% vs 86%, p<0.05; CpG2: 51% vs 65% p>0.05). Hypermethylation at the PPARγ promoter of plasma DNA correlated with changes in hepatocellular rather than myofibroblast DNA methylation. Similar results were demonstrated in patients with ALD cirrhosis.

**Conclusions:**

Differential DNA methylation at the PPARγ promoter can be detected within the pool of cell-free DNA of human plasma. With further validation, plasma DNA methylation of PPARγ could potentially be used to non-invasively stratify liver fibrosis severity in patients with NAFLD. Plasma DNA methylation signatures reflect the molecular pathology associated with fibrotic liver disease.

**Significance of this study**

**What is already known on this subject?**

- Non-alcoholic fatty liver disease (NAFLD) accounts for the majority of liver disease burden in the Western world.
- Liver biopsy remains the gold standard test to accurately stage fibrosis in patients with NAFLD, but it is invasive and carries risks.
- Differential DNA methylation of peroxisome proliferator-activated receptor gamma (PPARγ) has recently been shown to stratify fibrosis severity in liver biopsies of patients with NAFLD.

**What are the new findings?**

- Differential DNA methylation at the PPARγ promoter can be detected within the pool of cell-free DNA of human plasma, and may potentially stratify patients with NAFLD into mild versus severe fibrosis.
- Plasma DNA methylation signatures can be traced back to the molecular pathology in fibrotic liver tissue, providing a biomarker of the underlying pathological process.

**How might it impact on clinical practice in the foreseeable future?**

- This study demonstrates a novel, potential plasma biomarker of liver fibrosis and could, if validated, be used to non-invasively evaluate liver fibrosis severity, mitigating the future need for biopsy.

## Introduction

Non-alcoholic fatty liver disease (NAFLD) now comprises the majority of liver disease burden in the Western world. In particular, the prevalence of NAFLD is rising, in line with increasing prevalence of obesity and insulin resistance, as lifestyles have become increasingly sedentary and dietary patterns have changed.^1^ NAFLD is a spectrum of liver disease that includes simple steatosis, fatty infiltration plus inflammation and hepatocellular ballooning degeneration (non-alcoholic steatohepatitis; NASH), fibrosis and ultimately cirrhosis.^2^ Overall, NAFLD is associated with an increased risk of both cardiovascular disease and liver-related mortality.^3^ Recent studies indicate that both steatosis and NASH may progress to advanced liver disease^4–6^ and that the presence and severity of fibrosis is the key histological determinant of long-term prognosis;^7^ ^8^ this subset of patients are more likely to progress to decompensated cirrhosis, portal hypertension, hepatocellular carcinoma (HCC) and death without liver transplantation. Indeed, NAFLD is projected to be the primary indication for liver transplantation in many countries within a decade.^9^

It is imperative to accurately determine the presence of fibrosis in patients with NAFLD; a liver biopsy remains the gold standard for diagnosing and staging fibrosis, but is seldom performed due to perceived risk of complications. In addition, liver biopsy is subject to sampling error, while the pathology assessment of the degree of liver fibrosis and the stage of disease often suffers from interobserver error. Together, these problems have hampered both routine clinical care and the development of pharmacological treatments for fibrotic liver diseases. Non-invasive scoring systems with high negative predictive value, such as the NAFLD fibrosis score, are used in the clinic to exclude patients with advanced fibrotic disease.^10^ ^11^ However, up to one quarter of patients cannot be classified using this scoring system,^10^ and so require liver biopsy for histological clarification. Liver stiffness measurement (FibroScan/acoustic radiation force impulse) performs well for the diagnosis of liver fibrosis, but has a low success rate in obese patients with NAFLD.^12^ Ultimately, there is an urgent and unmet need to develop non-invasive markers that can accurately stratify fibrosis severity in patients, reflect underlying fibrotic processes, and could potentially be used to monitor disease progression and a therapeutic response to emerging antifibrotic medicines.

While there is unquestionably a role for gene polymorphisms in the progression of NASH to fibrosis there is also strong evidence for the involvement of lifestyle factors (age, diet, exercise, other comorbidities and so on).^1^ It is increasingly apparent that the environmental and lifestyle experiences of an individual are important influences on disease susceptibility and outcome. Furthermore, there is increasing awareness that these extrinsic experiences impact on gene expression through numerous epigenetic mechanisms, with the result that the phenotype and behaviour of cells and tissues are modified in a dynamic fashion. There is a steady accumulation of experimental and observational clinic information supporting the concept that epigenetic processes orchestrate the behaviour of liver cells and underpin the pathobiology of most liver diseases including NAFLD.^13^ DNA methylation is a fundamental epigenetic modification of DNA that occurs at the cytosine base within a cytosine-guanine dinucleotide (often referred to as CpG).^14^ A long-held biological dogma is that CpG methylation is a stable feature of the genome that is annotated during embryo development and serves to repress gene expression either by inhibiting the binding of transcription factors at gene promoters or by recruiting methyl DNA binding proteins that subsequently assemble gene transcription repression complexes.^15^ However, the recent discovery of mechanisms that operate to demethylate DNA and the development of next-generation sequencing protocols that allow sequence-specific quantification of DNA methylation have revealed that DNA methylation is not fixed but is highly dynamic and is responsive to cellular and tissue microenvironments.^16^ ^17^ Indeed the epigenetic evolution of cancer is now a well-established concept and can be tracked by monitoring methylome changes during the growth and spread of a tumour and in response to chemotherapy.^18^ Work in our laboratory has shown that dynamic changes in DNA methylation are mechanistically implicated in the pivotal event of fibrogenesis, the activation (or transdifferentiation) of hepatic stellate cells (HSCs) into matrix-producing myofibroblasts.^16^ ^19^ ^20^ Additionally, we have published observational studies using archival human NAFLD and alcoholic liver disease (ALD) biopsy tissues to demonstrate that differential methylation densities at a number of fibrosis-regulating gene loci (peroxisome proliferator-activated receptor gamma (PPARγ), PPARα, tumour growth factor beta and platelet-derived growth factor alpha (PDGFα)) can be employed to stratify fibrosis severity.^21^ ^22^ As an example, the PPARγ is a member of the nuclear hormone receptor family,^23^ involved in lipid storage and metabolism, glucose homeostasis and adipogenesis.^24^ ^25^ It is also a master negative regulator of HSC activation and liver fibrogenesis.^19^ Of relevance to the use of DNA methylation as a potential stratification tool, the promoter region of PPARγ undergoes methylation remodelling and becomes hypermethylated as fibrosis severity increases in human NASH liver biopsies.^21^ However, measurement of methylation at the PPARγ promoter in these studies has relied on access to liver tissue and hence is dependent on a biopsy.

The main aim of the present study was to ask if differential DNA methylation at the PPARγ promoter can be detected within the pool of cell-free DNA of human plasma and if so then do levels of methylation at this loci correlate with fibrosis stage in NASH.

## Methods

### NAFLD cohort

Use of human tissue was approved by Newcastle and North Tyneside Local Research Ethics (approval number H10/H0906/41). All samples were collected and used subject to patients’ written consent. Patients were identified from a subspecialist tertiary NAFLD clinic at the Freeman Hospital, Newcastle upon Tyne, UK. All liver samples were collected and used subject to patient's written consent prior to the day of surgery. Two patients transplanted for cirrhotic NAFLD from the Freeman Hospital, Newcastle upon Tyne, UK were included in the study. Liver biopsies were performed as part of investigation of abnormal liver function tests, or to stage disease severity, in patients with radiological evidence of NAFLD. Patients with alternate liver diagnoses or evidence of coexistent liver disease (haemochromatosis, viral hepatitis, Wilson's disease, α-1-antitrypsin deficiency or autoimmune liver disease) were excluded. Patients who consumed more than 20 g of alcohol per day for males or more than 10 g per day for females were excluded. Clinical and laboratory data were collected from the time of liver biopsy. Relevant clinical details such as gender, age, weight, height and average current and previous alcohol intake (g/day) were obtained from all patients at the time of liver biopsy. The body mass index was calculated by the formula: weight (kg)/height^2^ (m^2^). Patients were identified as having type 2 diabetes if they were receiving dietary, oral hypoglycaemic drug or insulin treatment for diabetes, or had fasting blood glucose >7.0 mmol/L or glucose >11.1 mmol/L following an oral glucose tolerance test. Blood tests taken at the time of liver biopsy were used to calculate the NAFLD fibrosis score as previously described.^26^

### ALD cohort

Use of human tissue was approved by Newcastle and North Tyneside Local Research Ethics (approval number H10/H0906/41). Blood was collected at the time of patient identification in the liver clinics at the Freeman Hospital, Newcastle upon Tyne, UK. All liver samples were collected and used subject to patient's written consent prior to the day of surgery. Four patients transplanted for cirrhotic ALD from the Freeman Hospital, Newcastle upon Tyne, UK were included in the study. A diagnosis of ALD was made with abnormal serum transaminases, the presence of excess alcohol intake (>60 g/day for males, >40 g/day for females) and the exclusion of other diagnoses such as viral hepatitis (HBV, HCV and HIV), hereditary hemochromatosis, Wilson's disease, autoimmune hepatitis, α1 antitrypsin deficiency and drug-induced liver injury.

### Histological assessment

Percutaneous liver biopsies were performed using a Menghini needle or an 18G BioPince liver biopsy system (Medical Devices Technologies, Gainesville, Florida, USA). Liver biopsies were all >15 mm in length and were interpreted by an experienced hepatopathologist (DT). Histological scoring was performed according to the NASH Clinical Research Network criteria.^27^ The NAFLD activity score was graded from 0 to 8 including scores for steatosis (0–3), lobular inflammation (0–3) and hepatocellular ballooning (0–2). Fibrosis was staged from 0 to 4.

### Control cohort

Use of human tissue was approved by Newcastle and North Tyneside Local Research Ethics (approval number H10/H0906/41). All samples were collected and used subject to patients’ written consent. Age-matched controls were identified from the general gastroenterology clinics and endoscopy services at the Royal Victoria Infirmary, Newcastle upon Tyne, UK. Patients had no signs or symptoms of liver disease, and no history of chronic illnesses.

### DNA methylation analysis

DNA was extracted from 200 μL of plasma using the QIAamp DNA blood mini kit and was bisulfite treated with EZ DNA Methylation Gold Kit (Zymo, USA) according to the manufacturer’s protocol. The bisulfite-treated DNA was eluted in 10 μL elution buffer.

Similarly, DNA was extracted from laser capture microdissected tissue (PALM MicroBeam, Zeiss, Germany) using the QIAamp DNA micro kit according to the manufacturer’s protocol and bisulfite treated as above. A detailed method is available in online supplementary documents.

10.1136/gutjnl-2016-311526.supp1 Supplementary data

### Pyrosequencing analysis

Methylation of specific cytosines within CpG dinucleotides was quantified by pyrosequencing using a Pyromark Q96 MD (Qiagen) instrument. PCR and sequencing primers were obtained from a custom-designed assay for PPARγ (Eurofins Genomics, Luxembourg) as previously described.^21^ Ten microlitres of biotin-labelled PCR product was used in each well and combined by streptavidin-coated sepharose beads, washed in 70% ethanol, denatured in 0.01% sodium azide and washed in a wash buffer (Qiagen, PyroMark Wash Buffer, 979008). Sequencing primers were annealed to DNA product at 80°C. Samples were run in duplicate. Assay efficiency was validated by unmethylated and methylated DNA (Qiagen, EpiTect PCR Control DNA Set, 59695). CpG methylation data were analysed by Pyro Q-CpG software 1.0.6.

### Statistical analysis

All statistical analyses were performed using SPSS software V.21.0 (SPSS, Chicago, USA). Continuous normally distributed variables were represented as mean±SD. Categorical and non-normal variables were summarised as median and range. χ^2^ test or Fisher's exact test was used to determine the distribution of categorical variables between groups. To compare the means of normally distributed variables between groups, the Student's t test was performed. To determine differences between groups for continuous non-normally distributed variables, medians were compared using the Mann–Whitney U test. The diagnostic performance of non-invasive tests was assessed by receiver operating characteristic (ROC) curves. The area under the ROC (AUROC) was used as an index to compare the accuracy of tests. The cut-off for diagnosis of advanced fibrosis was taken from the point of maximum combined sensitivity and specificity. The sensitivity and specificity for relevant cut-offs were also displayed.

## Results

A total of 26 patients with NAFLD were identified who had a liver biopsy to investigate abnormal liver enzymes or to stage fibrosis in those with imaging evidence of steatosis. Fourteen had mild fibrosis (Kleiner 0–2) and 12 had severe (Kleiner 3–4) fibrosis. The demographic and laboratory characteristics of all patients are shown in table 1.

**Table 1:** Characteristics of the NAFLD cohort

Clinical characteristic	Mild NAFLD fibrosis (F0–2) n=14	Advanced NAFLD fibrosis (F3–F4) n=12	p Value
Age (years)	57±7	59±12	0.56*
Gender (male)	29%	67%	0.052†
BMI (kg/m^2^)	36.0±5.5	36.0±7.3	0.996*
Diabetes	50%	67%	0.39†
ALT (IU/L)	55±37	62±19	0.55*
AST (IU/L)	39±13	53±12	0.01*
ALB (g/L)	46±3	45±4	0.37*
Platelets (×10^9^/L)	234±54	223±70	0.67*
AST/ALT ratio	0.80±0.23	0.91±0.27	0.29*
NAFLD fibrosis score	−0.87±0.95	−0.34±1.14	0.22*

Data expressed as mean±SD or median (range).

*Student's t test.

†χ^2^ test.

ALB, albumin; ALT, alanine transaminase; AST, aspartate transaminase; BMI, body mass index; NAFLD, non-alcoholic fatty liver disease.

### Plasma DNA methylation of PPARγ gene promoter increases with fibrosis severity in NAFLD

**Figure 1:**
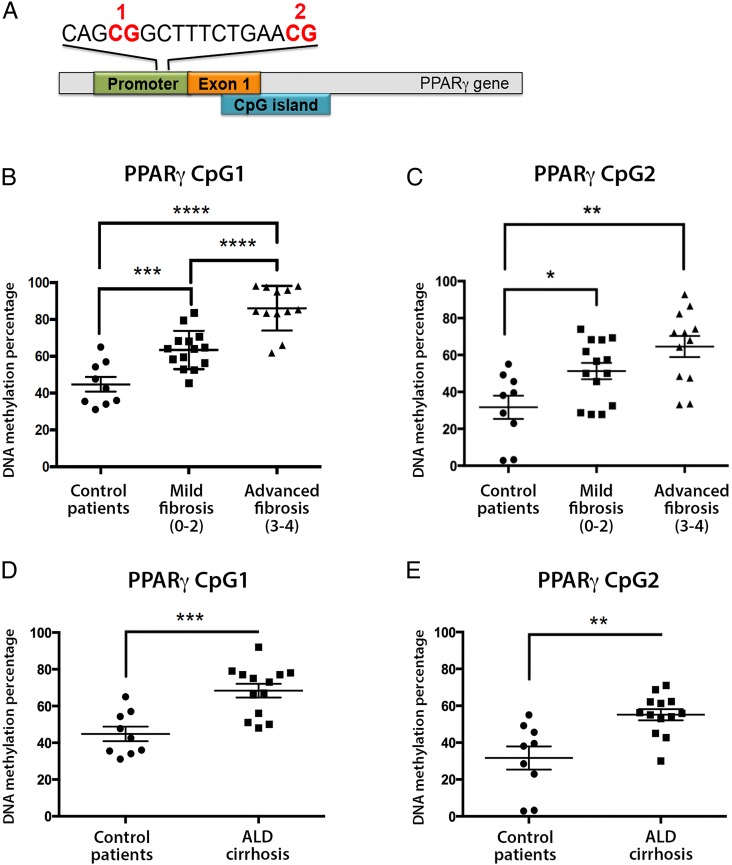
(A) Schematic representation of peroxisome proliferator-activated receptor gamma (PPARγ) gene promoter showing the positions of the differentially methylated CpG 1 and 2. (B and C) Plasma DNA methylation at (B) CpG1 and (C) CpG2 dinucleotide within the human PPARγ gene promoter from patients with mild or severe non-alcoholic fatty liver disease and controls as determined by pyrosequencing. (D and E) Plasma DNA methylation at (D) CpG1 and (E) CpG2 dinucleotide within the human PPARγ gene promoter from patients with cirrhotic alcoholic liver disease (ALD) and controls as determined by pyrosequencing. DNA methylation is quantitatively measured as expressed as a percentage. Error bars represent mean values±SEM. *p<0.05, **p<0.01, ***p<0.001 and ****p<0.0001.

In studies analysing DNA methylation in liver tissue, we have previously shown that differential DNA methylation at particular CpG dinucleotides within the human PPARγ gene promoter can be used to stratify fibrosis severity in patients with NAFLD.^21^ Quantitatively, in mild fibrosis, CpG methylation density was measured at >70% rising to >80% in severe fibrosis, at the two loci examined. In light of these results, it was logical to analyse the quantitative methylation of the same gene promoter region in PPARγ in plasma cell-free circulating DNA. To this end, we collected blood from patients and healthy controls, then isolated cell-free DNA (see online supplementary figure S1A). Using tested and optimised pyrosequencing primers (see online supplementary figure S1B), the amount of DNA methylation at two specific CpG loci within PPARγ promoter was quantitatively measured in all samples (figure 1A–C). We found increases in DNA methylation at both CpG loci in patients with severe versus mild fibrosis and controls (figure 1B, C), reaching high statistical significance between the patients with mild and severe NAFLD in the first CpG loci (figure 1B).

10.1136/gutjnl-2016-311526.supp2 Supplementary figures

Next, we wanted to assess whether increases in plasma DNA methylation at PPARγ gene promoter were fibrosis related or aetiology related. We therefore analysed 13 patients with clinical evidence of cirrhosis due to excessive alcohol consumption (ALD) and compared them with healthy controls, as it was not possible to find patients who had mild ALD. Clinical characteristics of the ALD cohort are shown in table 2. Remarkably, we again found evidence of fibrosis-associated hypermethylation at both CpG loci within the PPARγ promoter in patients with cirrhotic ALD when compared with healthy controls (figure 1D, E). These data suggest that PPARγ promoter hypermethylation is unlikely to be limited to a particular aetiology of liver disease but is instead closely associated with disease progression and development of fibrosis.

**Table 2:** Clinical characteristics of ALD cohort

Clinical characteristic	Cirrhotic ALD n=13
Age (years)	57±6
Gender (%male)	85%
ALT (IU/L)	34±14
AST (IU/L)	37±6
ALB (g/L)	39±6
Platelets (×10^9^/L)	136±61

ALD, alcoholic liver disease.

Finally, we analysed plasma DNA methylation at a specific loci at PDGFα gene; we chose PDGF as it is a well-established profibrotic gene, at which we have previously shown differential DNA methylation.^22^ In NAFLD liver biopsies, a particular CpG loci undergoes remodelling and becomes hypomethylated with increasing fibrosis severity.^22^ Remarkably, we found decreases in plasma DNA methylation at the same CpG loci in patients with NAFLD with severe versus mild fibrosis; similar decreases were found in patients with cirrhotic ALD versus controls (see online supplementary figure S2).

### Plasma DNA methylation of PPARγ as an independent predictor of fibrosis severity in NAFLD

**Figure 2:**
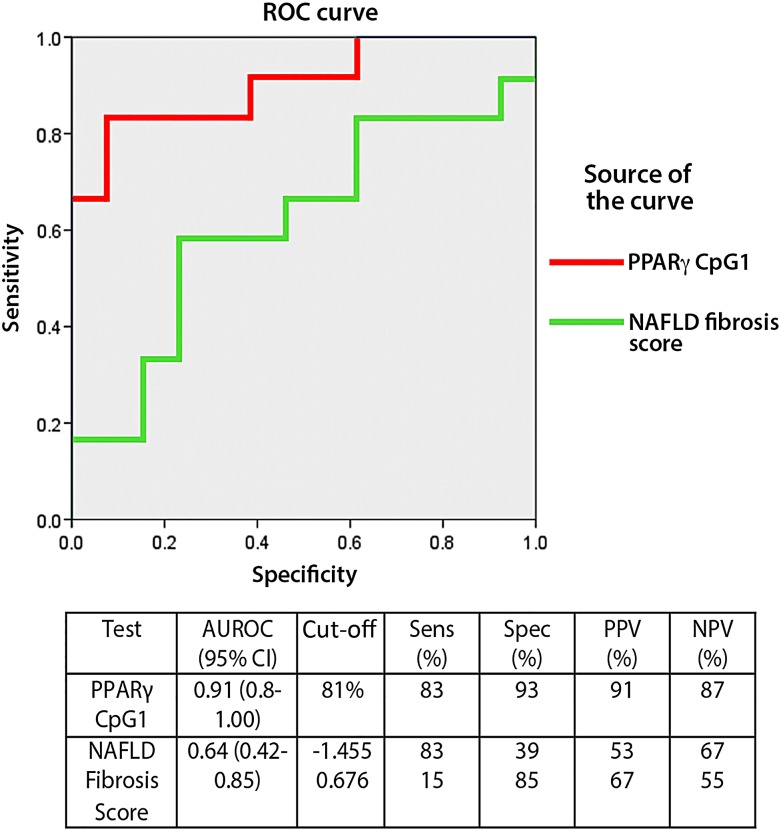
(A) Receiver operating characteristic (ROC) curves for the non-invasive diagnosis of advanced fibrosis (Kleiner fibrosis stage 3–4) using quantitative DNA methylation data for peroxisome proliferator-activated receptor gamma (PPARγ) CpG1 as compared with non-alcoholic fatty liver disease (NAFLD) fibrosis score. (B) A comparison of the performance of each test for the stratification of mild versus advanced fibrosis in 26 patients with non-alcoholic fatty liver disease (non-alcoholic steatohepatitis). AUROC, area under the ROC.

To ascertain whether plasma PPARγ DNA methylation level at locus CpG1 independently predicted fibrosis severity, we performed a multivariate logistic regression analysis. PPARγ DNA methylation level and clinical biochemistry indices found to be significant on univariate analysis (AST) were included to control for possible confounders of the association between fibrosis severity and plasma PPARγ DNA methylation level. PPARγ remained statistically significant (p=0.01), while AST lost significance (p=0.095). To assess the clinical applicability of this test we assessed its diagnostic performance for advanced NAFLD fibrosis (Kleiner 3–4) using the AUROC analysis (figure 2A). The AUROC was 0.91, which was signiﬁcantly higher than a chance assignment (asymptotic significance p=0.000). The threshold PPARγ CpG1 methylation value of 81% was the optimum cut-off to differentiate mild from advanced fibrosis. This score compared favourably with the NAFLD fibrosis score, a well-validated, widely used non-invasive fibrosis score (figure 2). The sensitivity, specificity, positive predictive value (PPV) and negative predictive value (NPV) for PPARγ CpG1 methylation and the NAFLD fibrosis score are shown in figure 2B.

### Hypermethylation at the PPARγ promoter of plasma DNA correlates with changes in hepatocellular rather than myofibroblast DNA methylation

**Figure 3:**
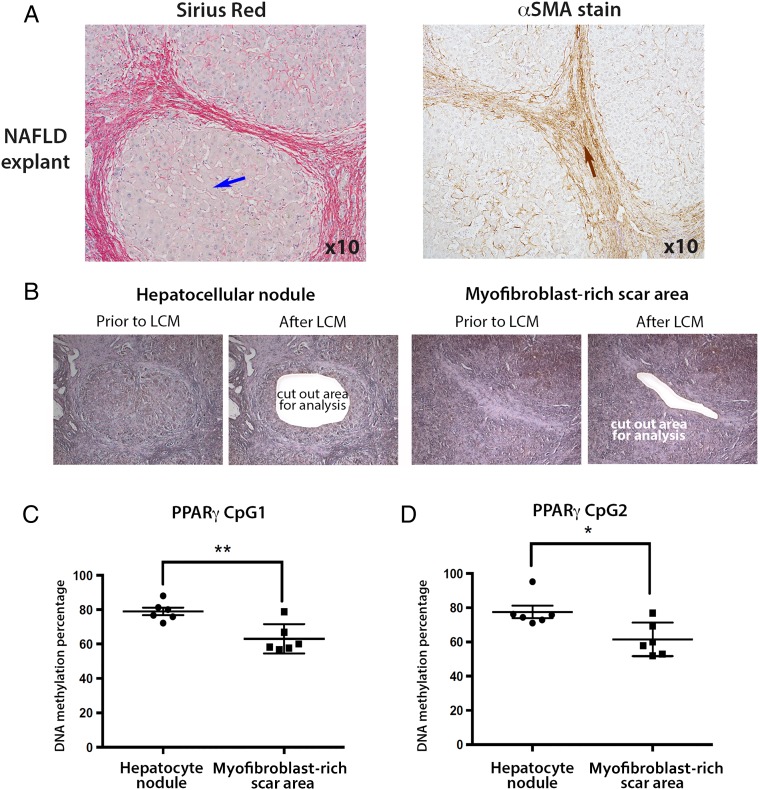
(A) Representative images of explanted non-alcoholic fatty liver disease (NAFLD) liver tissue stained with Sirius red and α-smooth muscle actin (αSMA). Blue arrow within Sirius red picture points to hepatocytes, whereas brown arrow within αSMA-stained section shows myofibroblasts in the scar region. Photomicrographs were taken at 10 times magnification. (B) Explanted NAFLD liver tissue was subjected to laser capture microdissection (LCM); areas of hepatocytes were separated from myofibroblast-enriched areas. H&E-stained tissue prior to and after LCM. (C and D) DNA methylation density of (C) CpG1 and (D) CpG2 dinucleotide within the human peroxisome proliferator-activated receptor gamma (PPARγ) gene promoter as determined by pyrosequencing in LCM material from NAFLD. DNA methylation is quantitatively measured and expressed as a percentage. Error bars represent mean values±SEM *p<0.05 and **p<0.01..

**Figure 4:**
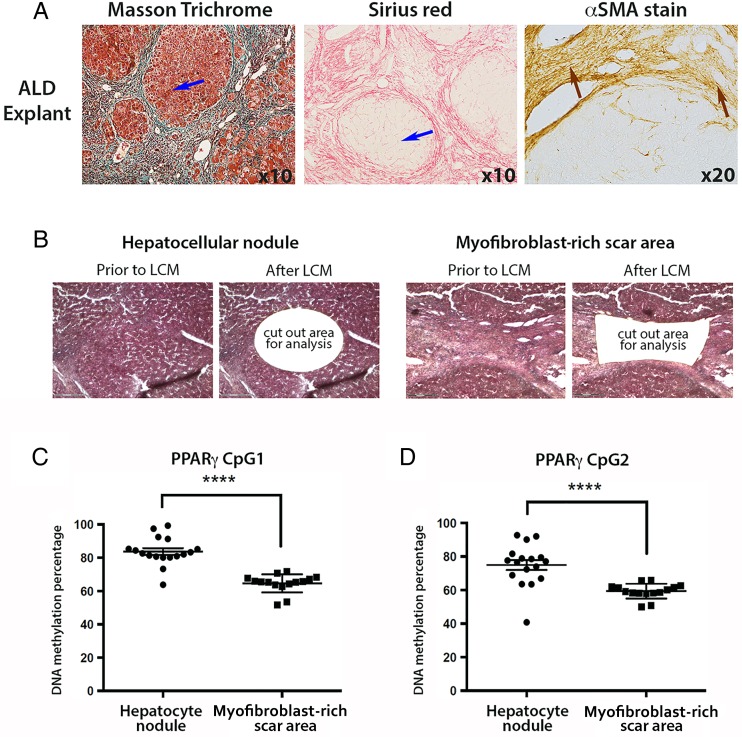
(A) Representative images of explanted alcoholic liver disease (ALD) tissue stained with Masson's trichrome, Sirius red and α-smooth muscle actin (αSMA). Blue arrows point to hepatocytes, whereas brown arrows point to the myofibroblasts in the αSMA stained scar region. Photomicrographs were taken at 10 times magnification. (B) H&E stained explanted ALD liver tissue was subjected to laser capture microdissection (LCM); areas of hepatocytes were separated from myofibroblast-enriched areas. Images show the tissue prior to and after LCM. (C and D) DNA methylation density of (C) CpG1 and (D) CpG2 dinucleotide within the human peroxisome proliferator-activated receptor gamma (PPARγ) gene promoter as determined by pyrosequencing in LCM material from ALD. The positions of the differentially methylated CpGs are shown in the schematic drawing above the graphs. Differences are expressed as a percentage of DNA methylation. Error bars represent mean values±SEM. ****p<0.0001.

We were next interested to determine if the degree of PPARγ promoter hypermethylation detected in plasma DNA reflected cellular changes in the diseased liver. In particular, we wanted to exploit the technology of laser capture microdissection (LCM) to separate myofibroblast-enriched fibrotic tissue from surrounding non-fibrotic hepatic parenchyma in advanced NASH liver explants. NAFLD explants were characterised by an expert histopathologist (DT) (figure 3A), then LCM was used to cut out either hepatocyte-rich regions (figure 3B, left panel) or myofibroblast-rich scar regions (figure 3B, right panel). Once again, the DNA methylation status of the two CpG loci within PPARγ promoter was determined by pyrosequencing using genomic DNA isolated from LCM-obtained tissues (figure 3C,D). Both CpG loci were hypermethylated in the hepatic parenchyma compared with areas enriched for myofibroblasts (figure 3C, D). The average DNA methylation percentages were 79 and 78 for CpG1 and CpG2, respectively, in the captured hepatocyte nodules compared with 63 and 61 in fibrotic tissue. When compared with similar measurements on parenchymal tissue from non-diseased liver tissue (76% and 70% for CpG1 and CpG2, respectively, online supplementary figure S3A), and control peripheral blood mononuclear cells (PBMCs) (68% and 63% for CpG1 and CpG2, respectively, online supplementary figure S3B), our LCM-pyrosequencing data suggest that PPARγ promoter hypermethylation detected in plasma more closely correlates with methylation changes in hepatocytes than in myofibroblasts or PBMCs. To corroborate this finding we carried out similar LCM-pyrosequencing assays using ALD explant tissues (representative images shown in figure 4A), again using LCM to isolate hepatocyte-enriched (figure 4B, left panel) or myofibroblast-enriched regions (figure 4B, right panel). As with NAFLD, we discovered relative hypermethylation of the CpG1 and CpG2 sites in the PPARγ promoter for hepatocyte-enriched versus myofibroblast-enriched tissues (figure 4C, D). Hence, irrespective of the cause of steatotic liver disease, progression to the advanced stage is associated with PPARγ promoter hypermethylation in DNA isolated from plasma and hepatocyte-enriched regenerative nodules.

## Discussion

Non-invasive or minimal-invasive biomarkers of disease progression are sorely needed in light of the currently rising liver disease burden. In particular, NAFLD is increasing in incidence, and is projected to be the primary indication for liver transplantation by 2020.^9^ Recently, the presence of fibrosis has been shown to be the key histological determinant of long-term prognosis.^7^ ^8^ Thus, an accurate determination of the degree of fibrosis is absolutely critical to allow effective management of this disease. The NAFLD fibrosis score is currently widely adopted in routine practice to help rationalise liver biopsy use, but for many patients fails to provide a satisfactory quantitative measure of their disease progression without referral to a liver biopsy.^10^ Dynamic biomarkers of disease progression that can be detected in patient blood offer the advantage of minimal-invasive regular monitoring of disease progression and if prognostic would be extremely helpful for clinical management and the design of clinical trials in NASH and other major forms of chronic liver disease.

Here we show in NAFLD and ALD that sequence-specific quantification of methylation densities at so-called differential DNA methylation regions (DMRs) may be employed to stratify patients according to their disease severity. Specifically, using two CpG sequences previously identified as DMRs in the PPARγ promoter we have shown that hypermethylation at these sequences correlates with advanced fibrosis/cirrhosis. This finding correlates with our previous data demonstrating that hypermethylation at the same PPARγ promoter DMRs in liver biopsy tissues can be used to identify patients who had progressed to Kleiner grade 3–4.^21^ While our study used only a relatively low number of patient samples (26 NAFLD and 13 ALD), the fact that PPARγ promoter hypermethylation correlated with disease progression across two distinct disease aetiologies is encouraging and supportive of further studies on the diagnostic and prognostic utility of PPARγ DMRs and indeed other genomic DMRs. Equally, our demonstration of differential plasma DNA methylation in two distinct and opposite gene DMRs further strengthens our study.

An interesting question is the cellular source of the hypermethylated PPARγ DNA in plasma of liver disease patients. Hepatocyte damage and death are characteristic components of the pathobiology of NASH and ALD and would be expected to result in substantial leakage of cell-free DNA into the circulation. Moreover, a recent paper has reported that the liver contributes significantly to the pool of DNA that circulates freely in the plasma.^28^ Given the similar degree of PPARγ hypermethylation at CpG1 and CpG2 in plasma and hepatocyte-rich tissue captured by LCM it is tempting to speculate that dying hepatocytes represent the cellular source of hypermethylated plasma DNA. However, at the time of writing we lack sufficient direct evidence to support this idea.

An unexpected finding from our LCM-pyrosequencing studies was a higher density of methylation at the CpG1 and CpG2 PPARγ promoter DMRs in hepatocyte-rich tissue compared with myofibroblast-rich fibrotic tissue, and this is observed in both NASH and ALD livers. There is strong association of PPARγ expression with the quiescent non-fibrogenic phenotype of HSC and a need to repress its transcription in order for the cells to adopt a myofibroblast phenotype.^29^ ^30^ We have previously shown that the methyl-CpG binding protein MeCP2 brings about this repression of PPARγ transcription at least in part by associating with methylated regions of the upstream promoter where it recruits chromatin remodelling factors.^19^ Hence, a higher degree of methylation at the PPARγ promoter would be expected to be a feature of fibrotic tissue in advanced liver disease. However, our comparison of non-fibrotic hepatocyte-rich tissue with fibrotic myofibroblast-rich tissue suggests that DMRs in the PPARγ promoter are more highly methylated in hepatocytes. Noteworthy is a recent cell-specific knockout study reporting that hepatocyte PPARγ1 mRNA is highly suppressed after experimental induction of liver fibrosis in mice.^31^ Experimentally, the loss of PPARγ expression in hepatocytes has been implicated as a signal for HSC preactivation and accelerated fibrogenesis^32^; such a process may constitute an important cross-talk mechanism between hepatocytes and HSCs that drives disease progression to fibrosis and cirrhosis. PPARγ has also been shown to have a regulatory role in hepatic regeneration. Mice with hepatocyte-targeted genetic deletion of PPARγ display impaired regeneration within the setting of diet-induced steatosis.^33^ Thus, hypermethylation of PPARγ in hepatocytes could also reflect impaired regeneration, a major characteristic of advanced liver disease and a driver of fibrosis.^34^ ^35^

There are a number of limitations with the current study. Clearly, the sample size is relatively small, with no independent validation within a separate cohort. Further, our method necessitates the use of prospectively collected fresh plasma samples, precluding the immediate use of archived plasma samples from existing cohorts at other centres. There is no data on the prognosis of patients enrolled in the study, and no longitudinal measurements, especially from interventional trials. Finally, the data does not suggest superiority to existing fibrosis biomarkers, although it performed favourably to the NAFLD fibrosis score in this cohort. However, given the ability to detect significant differential plasma DNA methylation in two separate gene DMRs in this small cohort of patients, patients with NAFLD fibrosis and patients with cirrhotic ALD, further work is warranted to validate these findings in a large cohort of patients with NAFLD, ALD and other fibrotic liver diseases.

In light of the limitations in the present study, there are clear directions for further research. First, a validation of the findings in a second independent cohort is required. Second, analysis of patients from an interventional trial with paired biopsies could reveal whether pathway-directed interventions (eg, the PPARα/δ agonist) alter the diagnostic performance of this marker. What we are at present unable to know is the degree to which DNA methylation at PPARγ and other genes carrying DMRs is changing during the disease course in an individual. Thus, the diagnostic relevance and the prognostic value of the biomarker should be assessed. Prediction of disease progression, during long-term follow-up, for early NAFLD stages using the novel technique should be evaluated. Furthermore, our knowledge of HCC-specific epigenomic signatures and its effect on the methylation pattern of PPARγ, when coexisting with NAFLD, should be assessed in future studies. Finally, a key question would be whether PPARγ hypermethylation is a preset feature of patients who are susceptible to disease progression or if it is a more dynamic modification that develops with disease.

In summary, our findings suggest that plasma DNA can be detected and potentially used to non-invasively stratify fibrosis risk in NAFLD according to methylation levels at DMRs within the PPARγ gene promoter. With validation, this blood-based biomarker could become an important contributory clinical tool alongside other epigenetic, genetic and biochemical biomarkers, mitigating the future need for biopsy to evaluate fibrosis.
