# Supplementary material for: Plasma DNA methylation: a potential biomarker for stratification of liver fibrosis in non-alcoholic fatty liver disease
Source: Gut. 2016 Mar 21;66(7):1321–8. doi: 10.1136/gutjnl-2016-311526 (PMC5031527; doi:10.1136/gutjnl-2016-311526)
Supplement: Supplementary data [file gutjnl-2016-311526supp001.pdf]

Supplementary Methods

Cell free circulating DNA extraction

Whole blood was collected into EDTA and the plasma was separated by centrifugation for 10 min at 3000rpm followed by transfer to new tubes and centrifugation for 10 min at 3000 rpm. The plasma was aliquotted and stored at -70 °C. Cell-free DNA was extracted from 200ul of plasma using QIAamp DNA Blood Mini Kit (Qiagen, Germany, catalogue no: 51106). Plasma was lysed at 56°C for 10 minutes, and the lysate was processed and transferred to spin columns as per manufacturer's instructions. DNA was eluted in 20ul buffer. The presence of cell free DNA was confirmed using a Bioanalyser (Agilent Biotechnologies, Santa Clara, USA) please see Supplementary Figure 1A. The total cf-DNA was measured using a Quant-iT TM DNA high-sensitivity assay kit and a Qubit fluorometer (Invitrogen, Carlsbad, CA, USA) in accordance with the manufacturer's instructions.

Bisulfite modification of cell free plasma DNA

Sodium bisulfite conversion was performed using EZ DNA Methylation Gold TM Kit (Zymo Research, Irvine, CA, USA). ~ 1ng cell free circulating DNA was bisulphite modified by incubating at 98°C for 10 min and 64°C for 2 h and 30 min. Product was transferred into columns; desulphonated and washed according to manufacturer's protocol and eluted in 10 µl of elution buffer. A 5 µl of bisulphite modified cell free

DNA was amplified in a PCR mix containing 2 µl of forward and reverse primer, 12.5 µl of HotStarTaq Master Mix Kit (Qiagen, Germany, catalogue no: 203445) and 5.5 µl of water. For PCR with PPARγ primers, 2.5ul Q solution and 1.5ul $MgCl_2$ (25mM/ml) were added and volume adjusted accordingly. For PCR with PDFGA primers, (HsAC147651.101 PM PyroMark CpG assay, Qiagen, PM00031745) no further additions were made and were used as per manufacturers guidelines. Amplification of DNA was performed in a thermocycler according to the following PCR conditions: one cycle at 95°C for 6 min, followed by 40 cycles of 95°C for 30 s, annealing temperature of 55°C for 30 s and 72°C for 30 s, followed by one cycle at 72°C for 30 s.

Laser Capture Microdissection- LCM of NASH liver explants

Tissue was collected at the time of surgery and was formalin-fixed, stained with hematoxylin and eosin (H&E) and Sirius red, and graded and staged for fibrosis by an expert hepatopathologist according to criteria published previously (12). For LCM, 10 µm thick sections were made onto glass slides, deparaffinised by treating with Clearene (Leica Biosystems, Germany), fixed in 70% ethanol, and hematoxylin-stained for morphologic and nuclear detail. LCM was performed using the Zeiss PALM MicroBeam; hepatic parenchyma was separated from myofibroblasts-enriched areas under direct visualisation. Four parenchymal segments and 4 fibrotic bands were cut per section according to anatomical landmarks. Each segment was between 1-2.5 million μm^2 in area. Suitable areas to laser microdissect were identified by an expert liver pathologist (DT) prior to LCM.

Tissue Preparation and LCM of ALD explants. LCM was performed as described. Four parenchymal segments and 4 fibrotic bands were cut per section according to anatomical landmarks. Each segment was between 1-2.5 million μm^2 in area.

DNA Extraction, Bisulfite Conversion and Pyrosequencing. DNA was extracted using QIAamp DNA Micro Kit (Qiagen, Germany, catalog no:56304). LCM material was lysed on 56°C overnight, treated with 70°C for 30 minutes to remove crosslinks that were formed by formalin and the lysate was processed and transferred to spin columns as per manufacturer's instructions. Genomic DNA was quantified using NanoDrop (Thermo Scientific, Nanodrop 2000, UV-Vis spectrophotometer). Sodium bisulphite conversion was performed using EZ DNA Methylation Gold TM Kit (Zymo Research, US). ~100ng of extracted genomic DNA from LCM samples was bisulphite modified by incubating at 98 ^{o}C for 10 minutes, 64 ^{o}C for 2 hours and 30 minutes. Product was transferred into columns; desulphonated and washed according to manufacturer's protocol and eluted in 10 µl of elution buffer. 5 µl of bisulphite modified genomic DNA was amplified in a PCR mix containing 2 µl of forward and reverse primer, 12.5 µl of HotStarTaq Master Mix Kit (Qiagen, Germany, cat no:203445) and 10.5 µl of water. Amplification of DNA was performed in a thermocycler according to the following PCR conditions: 1 cycle at 95°C for 6 minutes, followed by 50 cycles of 95°C for 30 seconds, annealing temperature of 55 ^{o}C for 30 seconds, and 72°C for 30 seconds, followed by 1 cycle at 72°C for 30 seconds.

Pyrosequencing- Methylation of specific cytosines within CpG dinucleotides was quantified by pyrosequencing using a Pyromark Q96 MD (Qiagen) instrument. PCR and sequencing primers were custom designed for PPARγ (Eurofins, Germany).

Human PPARγ pyrosequencing primers- forward primer GGAAAGAGGGGTTTTAAGTTTAGG; reverse primer CAATAACCTTTTCTTTTCCTACC; sequencing primer GGGGTTTTAAGTTTAGGAG. 10μl of biotin-labelled PCR product was used in each well and combined by streptavidin coated sepharose beads, washed in 70% ethanol, denatured in 0.01% sodium azide and washed in a wash buffer (Qiagen, PyroMark Wash Buffer, 979008). Sequencing primers were annealed to DNA product at 80°C. Samples were run in duplicate. Assay efficiency was validated with unmethylated (0% methylated) and methylated DNA (100% methylated) control DNA (Qiagen, EpiTect PCR Control DNA Set, 59695). CpG methylation data were analysed by Pyro Q-CpG software 1.0.6. Efficiency curves of PPARγ primers are presented in Supplementary Figure 1B.
