# Supplementary material for: Plasma DNA methylation: a potential biomarker for stratification of liver fibrosis in non-alcoholic fatty liver disease
Source: Gut. 2016 Mar 21;66(7):1321–8. doi: 10.1136/gutjnl-2016-311526 (PMC5031527; doi:10.1136/gutjnl-2016-311526)

Supplementary Figures

Supplementary Figure 1. A) Bioanalyser trace showing a peak of isolated cell free DNA. B) Human genomic DNA of known DNA methylation percentage was used as template to test the pyrosequencing assays. Results were plotted as known vs measured DNA methylation percentage.

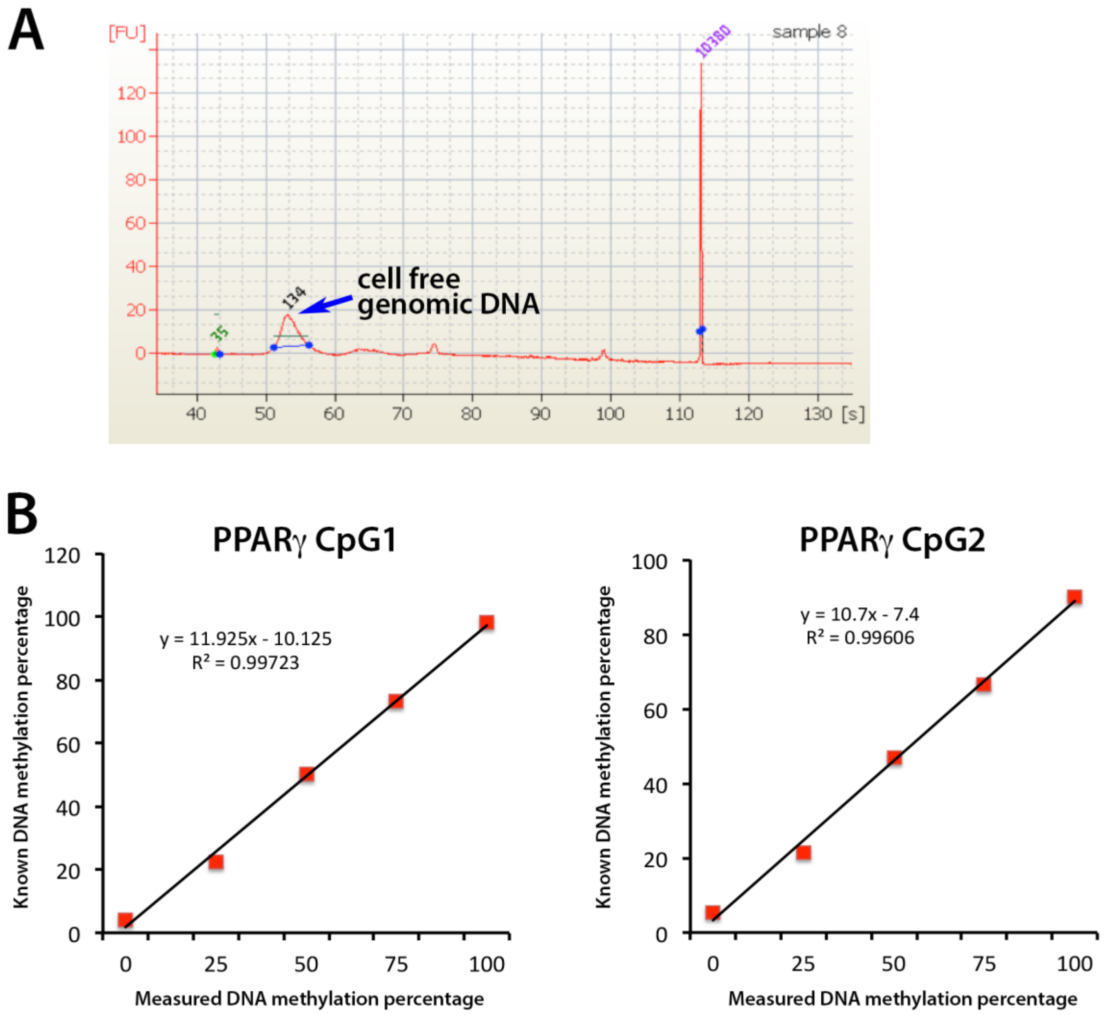

Supplementary Figure 2. A) Schematic representation of PDGFα gene promoter showing the position of the differentially methylated CpG. B-C) Plasma DNA methylation at CpG dinucleotide within the human PDGFα gene promoter from patients with (B) mild or severe NAFLD and (C) cirrhotic ALD and controls as determined by pyrosequencing. DNA methylation is quantitatively measured as expressed as a percentage. Error bars represent mean values ± standard error of the mean (SEM) *p<0.05. Four patients were excluded from the severe NAFLD cohort due to assay failure.

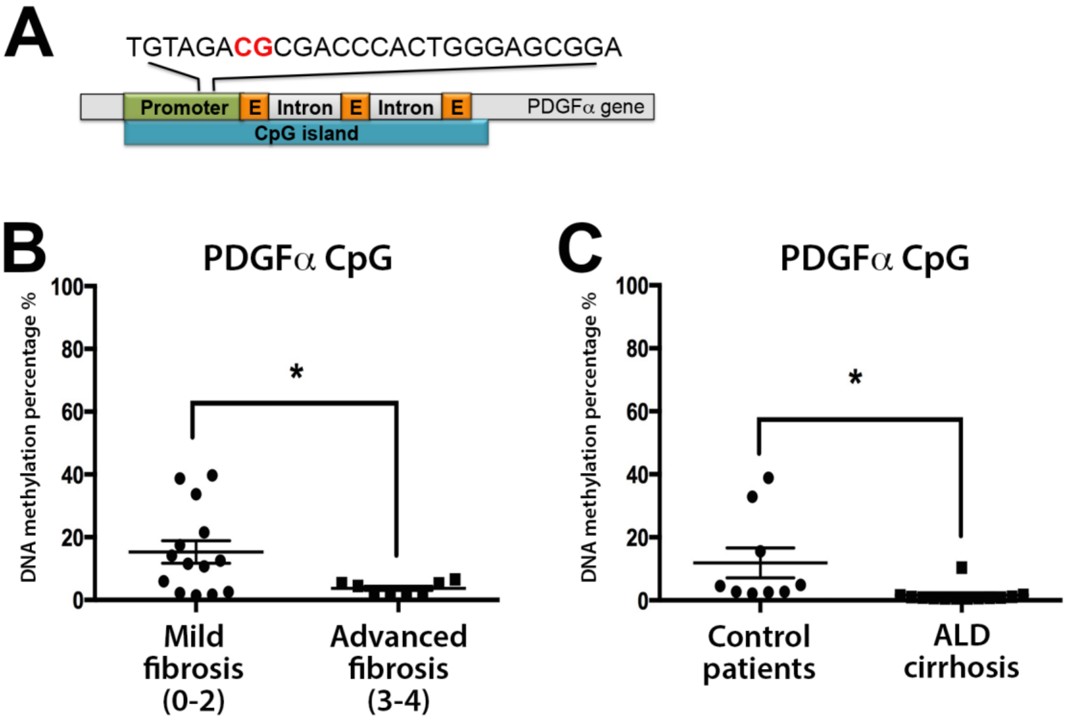

Supplementary Figure 3. A) Normal PBMCs were isolated and genomic DNA obtained. Normal hepatocytes were obtained using LCM in normal liver and genomic DNA isolated from LCM material. DNA methylation at CpG1 and (B) CpG2 dinucleotide within the human PPARγ gene promoter was determined by pyrosequencing. DNA methylation is quantitatively measured as expressed as a percentage. Error bars represent mean values ± standard error of the mean (SEM) *p<0.05, **p<0.01

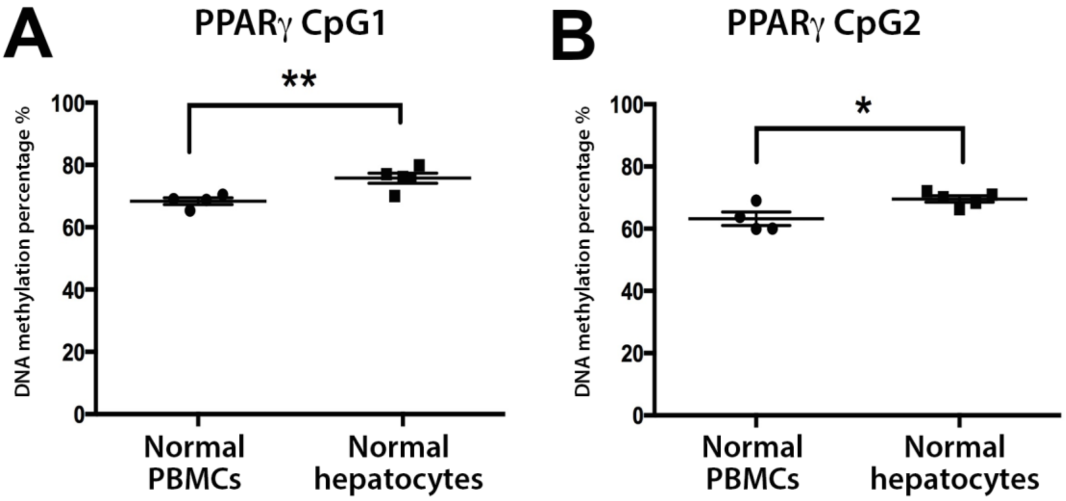

Supplement: Supplementary figures [file gutjnl-2016-311526supp002.pdf]
